# HIV-1 infection in polarized primary macrophages

**DOI:** 10.1186/1742-4690-8-S2-P11

**Published:** 2011-10-03

**Authors:** Viviana Cobos-Jiménez, Steven W de Taeve, Thijs Booiman, Karel A van Dort, Angélique B van 't Wout, Jörg Hamann, Neeltje A Kootstra

**Affiliations:** 1 Laboratory for Viral Immune Pathogenesis, Department of Experimental Immunology, Academic Medical Center, University of Amsterdam, Amsterdam, The Netherlands

## Background

Macrophages are important targets for HIV-1 infection and are involved in mucosal transmission of the virus. Due to their ubiquitous distribution, macrophages play a crucial role in virus spread and can become reservoirs for HIV-1. *In vivo*, macrophages are exposed to a multiplicity of signals that can polarize them into a classically activated M1 (IFN-γ, LPS and/or TNF-α) or into alternatively activated M2a (IL-4, IL-13) and M2c (IL-10, glucocorticoids) phenotype. Previous studies have shown that the susceptibility of macrophages to HIV-1 infection is regulated by type I interferons (IFN-α, IFN-β) and by the cytokines IL-4 and IL-10, however the mechanism underlying the latter has not been described yet. In this study, the expression levels of HIV-1 restricting cellular factors in the different types of polarized monocyte-derived macrophages (MDM) was analyzed and their role in HIV-1 susceptibility was investigated.

## Methodology

Monocytes were isolated from buffy coats from healthy blood donors using density gradient separation followed by plastic adherence. MDM with a M1, M2a and M2c phenotype were obtained by culturing cells for 5 days in the presence of IFN-γ, IFN-γ/TNF-α, IL-4 and IL-10, respectively. Monocytes were also stimulated with type I interferons and the colony-stimulating factors M-CSF and GM-CSF. Polarization of MDM was confirmed by flow cytometry. MDM susceptibility to infection was analyzed with HIV-1/NL4-3BaL and a VSV-G-pseudotyped luciferase reporter virus. Expression of HIV-1 host restriction factors was measured by RT-qPCR. Viral reverse transcription products were detected, in order to identify at which step inhibition of viral replication occurs.

## Results

Macrophages differentiated in the presence of IFN-α, IFN-β, IFN-γ ± TNF-α (M1), IL-4 (M2a) or IL-10 (M2c), differentially expressed characteristic membrane receptors, such as CD14, CD16, CD64, CD80, CD162, CD200R and CD206, confirming the activated/polarized phenotype. Unpolarized and M-CSF/GM-CSF-stimulated MDM were highly susceptible to infection, whereas IFN-α, IFN-β, IFN-γ±TNF-α, IL-4 or IL-10 treatment resulted in a significant inhibition of virus replication. Infection of these populations with a VSV-G-pseudotyped virus indicated that HIV-1 replication was inhibited at a post-entry level. Inhibition of viral replication occurs at an early step in the replication cycle, in MDM stimulated with type I interferons and in M1 and M2a MDM, whereas in M2c macrophages, inhibition occurs after reverse trannscription. Expression of HIV-1 restriction factors like APOBEC3G, Trim5α, CyPA, tetherin, Trim22 and recently identified anti-HIV miRNAs was upregulated in MDM treated with type I IFNs, and to a lesser extend in M1 polarized macrophages.

## Conclusions

These results suggest that the host factors analyzed here may contribute to inhibition of HIV-1 replication in MDM by type I interferons. However, these factors are not likely involved in HIV-1 inhibition in M1 or M2 macrophages. Additional studies are necessary to identify other host factors involved in the resistance of polarized macrophages to HIV-1 infection.

